# Ecotypic Variations Affected the Biological Effectiveness of *Thymus daenensis* Celak Essential Oil

**DOI:** 10.1155/2021/6686558

**Published:** 2021-02-09

**Authors:** Fatemeh Elahian, Maryam Garshasbi, Zahra Mehri Asiabar, Neda Gholamian Dehkordi, Alireza Yazdinezhad, Seyed Abbas Mirzaei

**Affiliations:** ^1^Department of Medical Biotechnology, School of Advanced Technologies, Shahrekord University of Medical Sciences, Shahrekord, Iran; ^2^Department of Pharmacognosy and Traditional Pharmaceuticals, School of Pharmacy, Zanjan University of Medical Sciences, Zanjan, Iran; ^3^Clinical Biochemistry Research Center, Basic Health Sciences Institute, Shahrekord University of Medical Sciences, Shahrekord, Iran

## Abstract

*Thymus* (Lamiaceae) is famous for its pharmacological properties. *Thymus daenensis* Celak (Avishan-e-denaee in Persian) is an endemic *Thymus* species in Iran and is traditionally used for its digestive, carminative, antitussive, antispasmodic, and expectorant attributes in folk medicine. Ecotypic oils were extracted and analyzed with the GC-MS. Their biological properties in terms of antimicrobial, antioxidant, and antigenotoxic activities were evaluated using the minimal inhibitory concentration, minimal bactericidal concentration, and DPPH, *β*-carotene, and comet assays. The GC-MS results for *Thymus daenensis* Celak oils revealed thymol (73.86%) and carvacrol (51.89%) as the most abundant components. Due to the results, reasonable bactericidal activity values range from 0.14 to 5.00 mg/ml, and fungicidal activity ranges from 0.17 to 0.58 mg/ml. The necessary oil free radical scavenging capacity (0.41–1.79 mg/ml), bleaching inhibitory activity (0.01–1.06 mg/ml), and genoprotective potential (1.04–7.78 mg/ml) indicated the dose-dependent activity. The results suggest that *Thymus daenensis* is an important antibacterial and antifungal bioresource. Additionally, the antioxidant and radical scavenging capacity suggests this species has a role as a natural preservative in oxidative diseases and in the prevention of food spoilage.

## 1. Introduction

Essential oils are complex natural and aromatic compounds, which are derived from different parts of the plant using extraction, fermentation, expression, and steam distillation. Terpenoids, phenolics, polyphenolics, polyacetylenes, lectins, polypeptides, and alkaloids are the main plant essential oil (EO) constituents, which can exhibit potent antibacterial, antiviral, antifungal, antioxidative, and cytotoxic properties. The wide spectrum of medicinal properties of herbal EOs medicinal make them strong candidates for use in the pharmaceutical, food, and cosmetic industries [1, 2].

Amongst the 928 species of *Thymus* or “Avishan,” which are widely used for medicinal purposes, *Thymus daenensis* or “Avishan-e-denaee” is one of the most important Iranian endemic species. The genus *Thymus* belongs to the family Lamiaceae (Labiatae), one of the best-known flowering plant families with approximately 236 genera [3]. *Thymus daenensis* comprises two known subspecies: 1. *T. daenensis* Celak subsp. *daenensis* and 2. *T. daenensis* subsp. *lancifolius*. *Thymus daenensis* is a valuable spice plant that occurs in the Zagros mountain chain and grows as a perennial dwarf shrub (6–30 cm high) with flowers and lanceolate leaves. *Thymus* phenolic and nonphenolic derivatives are mainly used to treat gastrointestinal disorders, nervousness, and headaches in the form of tonic or herbal tea. *Thymus* is an aromatic plant that contains monoterpenoid essential oils in its leaves and floral parts, similar to most species of the Lamiaceae. Previous research has also focused on the EO-mediated biomedical activities of other species of *Thymus* including antimicrobial, antioxidant, and also to less extent their antigenotoxic effects [4, 5].

The main concern about the medicinal plants is related to the environmental conditions effect on their medicinal activities diversity. Several studies have indicated that the climate, temperature, light, height above sea level, latitude, and longitude are the most important factors affecting the physical and biological behaviors of plants including their genetics, immunology, physiology, morphology, population divergence, and evolution properties [6]. Earlier studies highlighted the climate fluctuations influence on the EOs' composition of ecotypic *Thymus kotschanus* and as well examined biological variety quantitatively in their antimicrobial, antioxidative, and antigenotoxic behaviors [7]. At the first step of this study, we focused on the *T. daenensis* EO-mediated antimicrobial, antioxidative, and antigenotoxic behaviors. Then, climate and altitude effects on the EOs' composition of ecotypic *T. daenensis* and their biological diversity were investigated. In addition to the antimicrobial activity, the antioxidant and antigenotoxic activities were reported.

## 2. Materials and Methods

### 2.1. Reagents and Media

Butylated hydroxytoluene (BHT), 2,2-diphenyl-1-picrylhydrazyl (DPPH), polyunsaturated linoleic acid, and beta-carotene (*β*-Carotene) were provided from Sigma-Aldrich (Sigma-Aldrich, Deisenhofen, Germany). Low-melting agarose and Ficoll-Paque (density 1.077 g/ml) were attained from Thermo Fisher Scientific (Rockford, IL, US) and GE Healthcare (Madison, WI, USA), respectively. Luria-Bertani, Mueller-Hinton, and Sabouraud dextrose Media were purchased from Merck Company (Darmstadt, Germany). Also, other pure analytical grade compounds (>99% purity) were attained from the Iranian commercial resources.

### 2.2. Standard Bacterial and Fungal Microorganisms

Three pure lyophilized Gram-negative bacteria of *Pseudomonas aeruginosa* (ATCC 27853), *Salmonella typhimurium* (ATCC 13311), and *Escherichia coli* (ATCC 8739); three Gram-positive bacteria of *Enterococcus faecalis* (ATCC 29212), *Bacillus subtilis* (ATCC 12711), and *Staphylococcus aurous* (ATCC 25923), and also two fungi *Candida albicans* (ATCC 10231), and *Aspergillus niger* (ATCC 9142) were purchased from the “Iranian Research Organization for Science and Technology” (a member of the WFCC). Methicillin-resistant *Staphylococcus aurous* (MRSA) was a hospital-resistant sample and was devotedly provided by Professor Moghimi. Our department confirmed the purity and character.

### 2.3. Medicinal Plant Materials and Hydrodistillation

Fresh aerial parts of ecotypic *Thymus daenensis* Celak were collected from their habitation in Ghazvin, Isfahan, Markazi, Lorestan, and Zanjan provinces. After that was the identification of species; they were harvested from the proposed areas randomly, and the shades dried for two weeks at ambient temperature (20–25°C). As a part of an ecotypic morphology study, the fresh plant ecotypes macroscopic diameters were measured, and also images were recorded with a digital camera (COOLPIX P510, Nikon, Japan) equipped with GPS. Voucher specimens were recognized and deposited at the Herbarium of Department of Pharmacognosy, Faculty of Pharmacy, Zanjan University of Medical Sciences, Zanjan, Iran. The plants were subjected to hydrodistillation using a modified Clevenger's apparatus at 100°C for 3 hours. During the hydrodistillation process, plant materials were placed in boiling water. Essential oils could be easily separated from the collecting tube water phase. Anhydrous sodium sulfate was used as drying agent. The oils were stored in the opaque air-tight sealed bottles at 4°C for 3 months, until the time of physicochemical and biological tests running [8].

### 2.4. Physical Characterization of the Essential Oils

The yield was defined as the amount of essential oils formed (ml) related to the 100 g of consumed plant. The oil refractive indices and densities were measured using an Atago RX-7000a refractometer, and a liquid micro-pycnometer. The oils different serial concentrations were provided in methanol (1 : 2, 1 : 4, 1 : 8, 1 : 16, 1 : 32, 1 : 64, 1 : 128, 1 : 256 mg/ml), and absorption spectrum was recorded in absorbance range (230–1000 nm) using spectrophotometer (TECAN, Infinite®-M200; Austria). The oils molar absorptivity potencies against the UV light were attained using Beer's law (*A* = *εbC*, where *A*, *ε*, *b*, and *C* stand for absorbance, molar absorptivity, path length, and oil concentration, respectively) [7].

### 2.5. Oils Characterization by Using Gas Chromatography (GC-FID)

An HP-6890 series gas chromatography system was used for the *T. daenensis* oil analyses. This Agilent instrument was equipped with an HP-5 capillary column packed with 5% phenyl, and also 95% methylpolysiloxane (30 m × 0.32 mm ×  0.25 *μ*m capillary dimensions), and a flame ionization detector (FID). Nitrogen was applied as the carrier, and this gas flow was set on 1 ml/min. The column oven temperature was accurately set for 3 minutes at 60°C, and after that progressively increased to 250°C for 65 minutes. The injection port and the flame ionization detector were heated to 255 and 290°C, respectively. The sample split ratio was 1 : 10 [9].

### 2.6. Identification of the Oils Constituents Using GC-MS

Volatile constituents were identified using a gas chromatograph (Agilent, GC-7890A, USA) connected to an Electron Ionization Mass Spectroscopy, EIMS-5975C, detector (70 eV energy), and a capillary HP-5 MS well-packed column containing 95% of the methylpolysiloxane, and 5% of phenyl (dimensions: 30 m × 0.25 mm × 0.25 *μ*m). The central oven was preset for 5 minutes at 40°C. After that, the temperature was progressively increased to 230°C for 20 minutes, and at the final step, temperature was raised up to 280°C for 2 minutes (30°C/min ramping rate). Detector and injector temperatures were fixed at 240 and 250°C, respectively. One *μ*l of the oils was injected with a split ratio of 1 : 10 to the instrument, and also the helium flow rate was tuned to 1 ml/min. The GC-MS analysis was completed using various internal standards for components quantification. The oil components arrangement was conducted based on the retention index ratios along with the homologous normal alkane series retention times under the same running condition. Additional parameters, which were used for the composition description, were the Wiley7n.L library, NIST library, published data fragmentation model, and the authentic compounds mass spectroscopy. The results were expressed as percentages of the individual constituent whole peak area in association with the whole region of all standardized peaks in the chromatogram [10, 11].

### 2.7. Antibacterial Activities

The study of essential oils antibacterial activity was conducted using NCCLS (National Committee for Clinical Laboratory Standards) guidelines, and the broth microdilution method was applied for the reliable and reproducible results. Cultivated bacterial strains were subcultured on Mueller Hinton Broth (MHB) to an initial OD_600_ = 0.1, and incubated again for a few hours at 37°C to an OD_600_ = 0.5, and were used as the inocula after 1 : 200 dilutions in MHB (standardized at approximately 1 × 10^6^ CFU/ml). 100 *μ*l of the bacterial suspension was dispensed in a 96-well plate consisting of 100 *μ*l of serially diluted oils in MHB during the range of 0.01–5.12 mg/ml. Up and about 2.0% dimethyl sulfoxide (DMSO) was used as a co-solvent, and it does not alter the test organisms' growth. Negative controls included a standard bacterial suspension, and the DMSO maximum doses without any test components. A serial dilution (a range of 0.5–256 *μ*g/ml) containing pure ampicillin sodium salt was used as the standard antibacterial agent. Plates were agitated and incubated for 24 hours at 37°C. Subsequently, 20 *μ*l of MTT solution (0.2 mg/ml) was applied to each well and incubated for 30 minutes at 37°C. The lowest sample concentrations without any color changes were determined as the MIC (minimum inhibitory concentration). A 5 *μ*l portion from the colorless wells was subcultured on Mueller Hinton Agar (MHA) and was incubated for 24 hours at 37°C. MBC (minimum bactericidal concentration) was considered as the least oil concentration that killed ≥99.5% of the inoculated bacteria [12, 13].

### 2.8. Antifungal Activities

All of the essential oils ecotypes were examined for their fungal toxic activities individually, by an adapted broth microdilution assay. The *Aspergillus* was cultured in Sabouraud dextrose agar at 30°C for a week and also stored at 4°C for another week in order to spore germinating. The fungal spores were suspended in sterile normal saline solution supplemented with 0.1% v/v Tween-80, and isolated using several sterile cheesecloth aseptically layers. That flow through the spore suspension was counted using a hemocytometer and adjusted at 10^6^ spores/ml. 100 *μ*l of the conidial spore suspensions was added to 100 *μ*l of the essential oil serial dilutions from 0.01 to 5.12 mg/ml in Sabouraud dextrose broth. The plates were shaken for 30 seconds and after that incubated at 28 ± 0.5°C and in 80% relative humidity for 72 hours. Appropriate negative controls containing the standard fungal spore suspensions, and DMSO without the test components were produced, in order to confirm the sporangiospores viability, and any unwanted intrinsic effects absence. A fresh Sabouraud dextrose culture was diluted and adjusted to 5 × 10^6^ CFU/ml for *Candida albicans*, and 100 *μ*l of the suspension was dispensed in a 96-well plate containing 100 *μ*l of serially diluted oils and was cultivated for 48 hours at 28°C. The pure amphotericin-B serial dilution (0.1–12.8 *μ*g/ml) was used as the reference compound. MIC (minimum inhibitory concentration) was the least oil concentration and represented no visible fungal mycelia next to the incubation period. A 5 *μ*l portion was subcultivated on Sabouraud dextrose agar and was from the wells with complete fungal growth inhibition and incubated for 72 hours at 28°C. MFC (minimum fungicidal concentration) was considered as the lowest oil concentration that killed ≥99.5% of the inoculated fungi [14].

### 2.9. DPPH Method and Antioxidant Activities

The oil samples radical scavenging activities were evaluated against the discoloration of DPPH free radicals purple solution. In order to achieve this goal, 2 ml of prepared methanolic solutions of DPPH (80 *μ*g/ml) was mixed with the oils' methanolic dilutions equivalent volumes (final concentration: 0.01–1.28 mg/ml). The mixtures were incubated for 30 minutes in the darkness at ambient temperature. Absorbance values were recorded at 517 nm using a Tecan Infinite M200 spectrophotometer, and the oils free radical scavenging capacities (SC) were calculated according to the following equation:

(1) $$\begin{array}{c} \text{SC}\left(\%\right)=\frac{\left(A_{\text{NC}}-A_{\text{Sample}}\right)}{A_{\text{NC}}}\times 100. \end{array}$$

Negative controls (NCs) were composed of all the reagents excluding the antioxidant oils. In addition, reference antioxidants were examined like butylated hydroxytoluene and vitamin C (0.5 to 128 *μ*g/ml) in order to compare the compounds scavenging potencies. The relative scavenging activities were plotted against the essential oil concentrations, and the SC_50_ was considered as the essential oil concentration in order to result in 50% inhibition [15].

### 2.10. *β*-Carotene/Linoleic Acid Method and Antioxidant Activities

*Thymus* antioxidant potency was measured using the *β*-carotene bleaching inhibition in the linoleic acid presence. *β*-carotene/linoleic acid emulsion was prepared by mixing 1 mg *β*- carotene, 25 *μ*l of linoleic acid, and 200 mg Tween-20 in 1 ml of chloroform. The solvent was vacuum-evaporated using a rotary evaporator for 30 minutes at 40°C. After that, 100 ml of oxygenated water was composed with robust shaking, in order to form a stable emulsion. 350 *μ*l of the essential oil dilutions in ethanol (ranging 0.01–1.28 mg/ml) was mixed with 2.5 ml of the *β*-carotene/linoleic emulsion gently, at the final stage. All tubes were placed in a shaker incubator (50 rpm) for 2 hours, in the light and at 50°C. The absorbance difference was recorded for 2 hours, using a spectrophotometer against a negative control (NC), which contains all the reagents except oils at 470 nm. The beta-carotene oxidation inhibition percentage (BIC %) was calculated as follows:

(2) $$\begin{array}{c} \text{BIC}\%=\frac{\left[1-A_{\text{Sample}}0\;\min-A_{\text{Sample}}120\;\min\right]}{\left(A_{\text{NC}}0\;\min-A_{\text{NC}}120\;\min\right)}\times 100. \end{array}$$

Appropriate routine reference substances amounts were also applied including vitamin C and butylated hydroxytoluene (0.5 to 128 *μ*g/ml) for the antioxidant capacity evaluation. The relative antioxidative activities plot versus the oil concentrations was drawn, and BIC_50_ was measured from the best fitted line equation in the scatter plot [16].

### 2.11. Antigenotoxicity Activity (Comet Assay)

Comet assay is a highly sensitive method for studying of the compounds antigenotoxic activity against the DNA damage in lymphocytes. Lymphocytes were isolated from the volunteer blood samples using Ficoll-Paque TM PLUS2, and a cell suspension of 2 × 10^5^ cells/ml was prepared in the phosphate buffered saline. Then, 1 × 10^4^ cells were treated with the sampled oils serial concentration (0.01–5 mg/ml) for 30 minutes at 4°C. The cells were centrifuged and resuspended in 50 *μ*l of PBS and were finally mixed with the equal preheated low-melting-point agarose volume (1.5% w/v). By passing 30 minutes from incubation in ice, the cells were coated again with 100 *μ*l of low-melting agarose (0.75% w/v) in order to form a flat sandwich. After that, cell lysis was performed using ice-cold lysis buffer for 4 hours (90 mM EDTA, 2.25 M NaCl, 0.7% w/v NaOH, 9 mM Tris base, 1% v/v Triton X-100, 10% v/v DMSO; pH = 10). Then, the slides were neutralized and electrophoresed on ice under the alkaline conditions (1 mM EDTA, 300 mM NaOH; pH ≥ 13) at 0.30 A for 45 minutes. At the next stage, the microgels were neutralized, stained, and pictured with neutral Tris-buffer, ethidium bromide, and fluorescent microscope, respectively. DNA fragmentation was scored using Tritek CometScore13^TM^ software, and ([tail DNA/(head DNA + tail DNA)]×100) equation was applied for the tail DNA% determining. CIC_50_ is the sample concentration, which protects 50% genomic DNA from fragmentation using the scatter plot equation [7].

### 2.12. Statistical Analyses

All procedures were repeated three times independently, and their results were reported as the mean values ± the mean standard error. Quantitative data was statistically interpreted using IBM SPSS-22 software. Differences between experimental groups were determined using one-way ANOVA with Tukey's post hoc test. *P* values less than 0.05 were considered as statistically significant.

## 3. Results

### 3.1. Geographical Effects on the Physicochemical Properties of the Ecotypes

Different morphological analyses *of Thymus daenensis* ecotypes indicated a microenvironmental conditions distinct effect including altitude, humidity, and temperature on the plant height and also the total dry matter. This flowering plant is growing up to 35 cm tall with short trichomes on its leaves. The isolation process yields the yellowish oil with different productivity (0.36–0.86% v/w), based on the *Thymus* geographical origins. In addition, other ecotypic oils physical properties presented diversity including density and molar extinction coefficient at 280 nm, and it seems that they can also be directly affected by environmental conditions (Table 1).

### 3.2. Essential Oil Constituents in the *Thymus* Ecotypes

Sixty-one compounds were identified by the GC/MS analysis, in different *T. daenensis* ecotypes, and were representing 95 up to more than 99% of the total oil compositions. As displayed in Table 2, thymol (0.31–73.86%), carvacrol (5.14–51.89%), linalool (0.3–22.49%), pulegone (up to 17.57%), geraniol (2.02–14.84%), *α*-terpineol (0.58–14.01%), p-cymene (0.12–11.19%), o-cymene (5.19–8.05%), geranyl acetate (0.16–6.11%), neomenthol (up to 5.69%), *β*- caryophyllene (1.81–4.97%), terpineol (0.59–4.68%), and borneol (0.01–4.04%) are the major constituents in the *T. daenensis* volatile oil. Some compounds were only detected in special ecotypes, and they are identified as *δ*-3-carene, *δ*-elemene, *α*-farnesene, *α*-himachalene, isoborneol, isopulegol, Z-jasmone, Z-linalool oxide, 1-menthol, neomenthol, menthone, *α*- muurolene, piperitone, pulegone, and thymol acetate.

### 3.3. Antimicrobial Activities of Essential Oils

The MIC and MBC values for the *T. daenensis* oils are listed in Table 3. Generally, Gram-positive bacteria indicated high sensitivity against the *T. daenensis oils* antibacterial effects (*P* < 0.001). *Pseudomonas aeruginosa* and *Salmonella typhimurium* are the most resistant and sensitive strains amongst the Gram-negative strains, respectively. *Staphylococcus aurous* and *Bacillus subtilis* indicated the most resistance and sensitivity against the oils antibacterial effects, in the Gram-positive strains. TD5, TD3, and TD10 extracts were the most potent antimicrobial oils, while TD2 was the least bioactive oil against all of the bacterial strains. Additionally, diversity in oils compositions led to differences in antifungal activity. The MIC values of *T. daenensis* extracts for *Candida albicans* and *Aspergillus Niger* were 0.13–0.58 mg/ml and around 0.08–0.1 mg/ml respectively. The MFC spectrum of the *T. daenensis* methanol extracts against the *Candida albicans* (0.13–0.58 mg/ml) and *Aspergillus Niger* (0.16–0.7 mg/ml) was determined using the adapted broth microdilution assay (Table 4).

### 3.4. Antioxidative and DNA-Protecting Activities

Hydroperoxides presence can be spectrophotometrically evaluated using the beta-carotene/linoleic acid method. TD8 was the weakest bleaching inhibitor, but other capacities of this oil were remarkable and almost equal to the BHT or ascorbic acid (*P* > 0.05). In contrast, hydrogen donor compounds are strong antioxidants and also associated with the DPPH-H formation.  Table 5 represents the *T. daenensis* oils hydrogen donating potency. Although TD5 and TD6 presented similar and the most DPPH scavenging potency (*P* < 0.01),TD2 was the weakest antioxidant (*P* < 0.001) (Supplementary Figures S1 and S2). According to the comet assay analysis and also attained CIC_50_ (1.04 to 7.78 mg/ml), it can be concluded that the TD10 and TD9 would exhibit promising inhibitory effect on DNA damage and closer antigenotoxic activity versus BHT and ascorbic acid. The oil protecting properties on DNA rates as the following order: TD10 > TD9 > TD1 > TD3 > TD2 > TD5 > TD7 > TD8 > TD6 > TD4 (Table 5 and Supplementary Figure S3).

## 4. Discussion

Natural plant products were the most of the medicinal agent source traditionally and were used in order to provide enthusiasm for industrial drug discovery. *Thymus* genera are famous in folk medicine. The ecotypes of *T. daenensis* essential oils compositions have been evaluated, and approximately 62 constituents were identified in order to form 95–100% of the total volatile oil spectra, generally. Thymol and carvacrol were the main constituents with average amounts of 45.5% and 16.5%, respectively. Table 2 indicates that the oxygenated compounds (>80%) are more dominant in comparison with the hydrocarbon constituents (<20%). Oxygenated monoterpenes were the most abundant (60–84%) consisting of carvacrol, linalool, terpineol, and thymol. Monoterpene hydrocarbons, oxygenated sesquiterpenes, and sesquiterpene hydrocarbons were present in low amounts amongst the ecotypes.

The results demonstrated that the total phenolics contents strongly depended on the plant species, geographical origin, and genetic content. Tables 1 and 2 are in accordance with the earlier research data, which stated that the high temperature with low altitude would increase the phenolic components (thymol and carvacrol) ratio in the oils [17]. In addition to these observations, it was also found that the ecological properties like high relative humidity would significantly increase the nonphenolic terpenes (linalool) and noncyclic monoterpenes (geraniol) ratios in some ecotypes. Despite these correlations, some components of *T. daenensis* oils variation could not be interpreted only by the environmental conditions, and other factors like genetic background may affect the type and amount of the constituents. For instance, this compound was not detected in other ecotypes, even those with similar environmental conditions, while the content of noncyclic monoterpenes pulegone has increased in TD2 as an ecotype growing in high temperature [18].

Due to the other researches, while TD2 with the lowest phenolic (thymol and carvacrol) content, near 21%, indicated the weakest antibacterial effect in all strains, TD3, TD5, and TD10 were the most effective antibacterial oils with high phenols levels (74%, 80%, and 82%, respectively). The microorganism susceptibility to essential oil derivatives is also microorganism-dependent. As a conclusion, these phenolic components hydrophobic properties enable them to partition with the bacterial cell membrane lipids, and lead to disturbing the cell integrity, and also rendering them more permeable. Alkyl groups, hydroxyls, or aldehyde moieties would influence the phenols antimicrobial activity, possibly through interfering with cell wall enzymes and reducing the surface tension [19, 20].

Although *Aspergillus niger* was more sensitive to the oils cytostatic effect (*P* < 0.001), but the *T. daenensis* ecotypes indicated similar fungicidal effect on both fungi strains (*P* > 0.05). Based on the MIC data, the differences between the oils' cytostatic effects on the *Aspergillus Niger* were not statistically significant (*P* > 0.05). However, MFC data indicated that TD9 had less fungicidal effects in comparison with the other ecotypes (*P* > 0.05). TD2 and TD1 were the less bioactive agents against *Candida albicans* (*P* < 0.001), while TD4 and TD5 presented the most antifungal effects against *Candida albicans* (*P* < 0.001). Consistent with many publications, variations in the fungicidal effects were not consistent with the ecotypic oils phenolic contents; for example, TD1 with high thymol and carvacrol (68%) content presented a similar and even less antifungal activity compared to the TD2, TD7, TD8, and TD9 [21–23].

Large natural polyphenols are ordered from simple to complex molecules according to the phenol rings and structural ingredients number and arrangement [17]. They act as antioxidant agents by donating hydrogen from their phenolic hydroxyl groups in the food industry. It is believed that the hydroxyl moieties number, position, and the degrees of glycosylation, esterification, and polymerization would determine the polyphenol components' antioxidant activity. According to many researches, thymol and carvacrol are the most effective constituents in lipid peroxidation. A survey conducted on the different *Thymus* species has presented that the effective antioxidant derivatives' order is as follows: thymol > carvacrol > terpinene > myrcene > linalool > cymene > limonene > cineole > pinene [24, 25]. DPPH radical scavenging activity and *β*-carotene supplementation results also revealed that the *T. daenensis* ecotypes exhibited dose-dependent antioxidants scavenging properties, acting as free radical terminators. TD2 represented the highest SC_50_ and BIC_50_ value, due to the fact that it contains the least effective phenolic antioxidants percent. Although some researchers reported that phenolic compounds' high concentrations might induce DNA single/double strand breaks, this study's results indicated that the *T. daenensis* oil thymol and carvacrol contents could act as oxidative DNA damage effective inhibitors [26]. In this regard, TD10 were the best antigenotoxic extracts and TD2 with the lowest phenolic compounds level represented the least DNA protection ability.

## 5. Conclusion

The *Thymus* essential oil chemical composition was affected by plant microenvironment variation directly. In conclusion, the most biological aspects regarding *T. daenensis* antimicrobial, antifungal, and antioxidant activities are associated with the oil constituents. Although direct correlations between the major constituents and biological activities have not been found in some cases, the total phenolic contents are responsible for most behaviors. It seems that the presence of some trace components in the oils may demonstrate severe antagonistic or synergistic behaviors and lead to unpredictable biological functions.

## Figures and Tables

**Table 1:** Environmental conditions and physical properties of *Thymus daenensis* essential oils.

Ecotype	Ecotype location	City, province	Altitude (m)	Temperature (°C)		Average humidity (%)	Density (g/ml) at 25°C	*ε*280 nm (l/(cm × g))	Refractive index at 25°C	Yield (v/w %)
				Max	Min					
TD1	34 59 17N	Gharechaie highlands, Marekazi	1900	23.9	6	40	0.94 ± 0.12	2.83 ± 1.01	1.32 ± 0.01	0.82 ± 0.21
	49 19 49E									

TD2	33 15 00N	Khoramabad, Lorestan	1830	38.8	−1.6	57	0.84 ± 0.04	1.98 ± 0.77	1.45 ± 0.08	0.36 ± 0.28
	48 30 00E									

TD3	33 25 00N	Khoramabad, Lorestan	1900	38.8	−1.6	57	0.98 ± 0.05	7.41 ± 2.32	1.38 ± 0.03	0.56 ± 0.13
	48 40 00E									

TD4	39 52 42N	Daran, Isfahan	2500	23	−3.1	42	0.96 ± 0.11	3.22 ± 1.74	1.39 ± 0.06	0.60 ± 0.23
	50 23 21E									

TD5	34 05 06N	Kharkan, Markazi	1965	35.5	−4.4	31	0.88 ± 0.04	5.38 ± 0.82	1.44 ± 0.03	0.62 ± 0.17
	49 24 50E									

TD6	34 11 38N	Arak, Markazi	2404	27	3	46	0.93 ± 0.06	2.92± 0.19	1.46 ± 0.09	0.86 ± 0.25
	49 29 41E									

TD7	32 55 52N	Fereidonshahr, Isfahan	2615	21.3	−4.5	50	0.88 ± 0.02	7.99 ± 1.58	1.51 ± 0.10	0.59 ± 0.23
	50 07 01E									

TD8	33 46 24N	Robatkhomein, Markazi	2407	25.2	3	51	0.89 ± 0.08	11.43 ± 1.63	1.47 ± 0.04	0.69 ± 0.12
	49 52 57E									

TD9	36 33 15N	Zanjan, Zanjan	1950	32.1	−7.5	54	0.90 ± 0.07	6.10 ± 1.94	1.51 ± 0.03	0.74 ± 0.26
	48 22 14E									

TD10	36 30 00N	Chore, Ghazvin	1950	18	2	51	0.95 ± 0.03	2.66 ± 0.19	1.48 ± 0.06	0.75 ± 0.11
	49 43 00E									

**Table 2:** Essential oil compositions for ecotypic *Thymus daenensis* (%).

Compounds	RI	RI*∗* [11]	TD1	TD2	TD3	TD4	TD5	TD6	TD7	TD8	TD9	TD10
*α*-Pinene	938	939		0.86	0.42	0.26				0.83	0.10	
Camphene	953	954		0.14	0.08					0.36		
*β*-Pinene	978	979		0.58						0.26	0.29	
1-Octen-3-ol	980	979		0.08	0.23					0.18		
*β*-Myrcene	990	991	0.81							0.70		
*α*-Terpinene	1017	1017	0.06				0.46			2.80		0.75
p-Cymene	1025	1026	0.12	2.56	5.12	6.53		11.19	0.23	9.26	1.64	0.38
O-Cymene	1026	1027		5.19			8.05					
*δ*-3-Carene	1029	1031		0.12								
1,8-Cineole	1030	1032	0.10	1.76	2.76	1.08	0.40	0.92	0.18	1.73	0.13	0.21
Z-*β*-Ocimene	1037	1038	0.20									
E-*β*-Ocimene	1047	1050							0.21		0.18	
*γ*-Terpinene	1059	1060	0.03	0.44	1.78	0.66	1.40	1.68		3.91	0.26	
Z-Linalool oxide	1074	1075	0.34									
*α*-Terpinolene	1087	1089				0.13				0.41		
Linalool	1098	1097	1.57	18.24	1.48	1.02	1.60	0.44	22.49	0.32	2.01	0.30
Z-*β*-Terpineol	1144	1144			0.16		0.60	0.24		0.59	0.12	
Camphor	1148	1150		2.88	0.26				0.48	0.62		
Menthone	1152	1153		2.22								
Isopulegol	1150	1152		2.12								
Isoborneol	1161	1162										0.62
Neomenthol	1167	1168		5.69								
Borneol	1169	1170	2.85	1.99	2.44	3.99		1.44	1.64	4.04	0.69	0.01
Terpinen-4-ol	1175	1177	1.04	0.60	4.68	0.70		0.59	1.10	0.90		
Isomenthol	1181	1183		3.00								
*α*-Terpineol	1189	1190	1.92		0.58	1.62		0.58		2.28	14.01	0.61
Thymol methyl ether	1230	1235			0.22	0.12						
Pulegone	1234	1237		17.57								
Carvacrol methyl ether	1243	1245	3.14					1.90				
Piperitone	1249	1253		1.97								
Geraniol	1253	1254	2.02								14.84	
Thymol	1303	1304	60.61	0.31	58.94	64.52	70.95	60.60	0.73	39.45	25.00	73.86
Carvacrol	1308	1310	7.45	20.90	14.86	5.14	8.82	6.22	51.89	16.29	26.12	7.89
*δ*-Elemene	1337	1339		0.08								
Piperitenone	1340	1343		1.90								
*α*-Terpinyl acetate	1347	1349	1.10			0.08						0.41
Thymol acetate	1354	1352								0.15		
Eugenol	1358	1359	0.39									0.05
*α*-Copaene	1376	1377								0.24		
Geranyl acetate	1381	1381	1.85				0.16		6.11		0.94	
*β*-Bourbonene	1389	1388	0.14	0.44	0.04	0.04			0.12	0.12	0.14	
*β*-Elemene	1390	1393		0.08								
Z-Jasmone	1393	1395	0.14									
Β-Caryophyllene	1420	1419	4.56	3.73	3.08	4.60	1.81	2.32	3.50	4.97	3.40	3.60
Aromadendrene	1440	1441	0.16					0.20		0.53		
*α*-Himachalene	1451	1452				0.35						
*α*-Humulene	1456	1455	0.24		0.12	0.17	0.10	0.11		0.24	1.15	0.20
*γ*-Muurolene	1480	1482	0.31		0.22		0.29	0.25		0.39		0.78
Germacrene D	1481	1485		0.21					0.75		0.20	
Viridiflorene	1492	1497	0.46			0.26		0.47		0.62		0.51
*α*-Muurolene	1501	1500		0.28								
*α* -Farnesene	1504	1506							0.34			
*β*-Bisabolene	1507	1506	1.47		0.40	0.82	0.67	2.65	0.83	0.66	0.85	1.24
Cis-*α*-Bisabolene	1508	1507	0.66	0.26	0.23	3.82	0.11	1.68	0.20	1.33	2.68	4.61
*γ*-Cadinene	1515	1514	0.41	0.33	0.26		0.16	0.15	0.15	0.28		
*δ*-Cadinene	1524	1523	0.41	0.28	0.52		0.31	0.44		0.59		0.66
Spathulenol	1579	1578	0.38	0.65		0.37		0.26	0.21	0.46	0.32	0.36
Caryophyllene oxide	1584	1583	0.27	0.41	0.38	1.84	0.71	0.84	0.93	2.00	1.69	1.56
Cubenol	1648	1646		0.10					0.52			
*α* -Cadinol	1655	1656	0.08	0.72					3.58	0.06		
*α*-Bisabolol	1687	1685								0.06		0.11
Others	—	—	4.71	1.31	0.74	1.88	3.40	4.83	2.81	2.37	3.24	1.28

Results are the average of two independent GC experiments. In order to reduce table complexity, the standard deviation values were ignored. RI represents the experimental retention index and RI*∗* donates the published retention index, extracted from Adams book [11].

**Table 3:** Minimum inhibitory concentration (MIC, mg/ml) and minimum bactericidal concentration (MBC, mg/ml) values for ecotypic *T. daenensis* essential oils.

Bacteria		Ampicillin	TD1	TD2	TD3	TD4	TD5	TD6	TD7	TD8	TD9	TD10
*Escherichia coli* (Gram-negative)	MIC	0.002 ± 0.00^a^	0.29 ± 0.00^b,c,d^	0.39 ± 0.15^c,d^	0.15 ± 0.00^a,b^	0.15 ± 0.00^a,b^	0.21 ± 0.08^a,b,c^	0.44 ± 0.17^d^	0.1 ± 0.08^a,b^	0.14 ± 0.00^a,b^	0.28 ± 0.00^b,c,d^	0.30 ± 0.00^b,c,d^
	MBC	0.064 ± 0.00^a^	0.58 ± 0.00^b^	0.79 ± 0.30^b^	0.15 ± 0.00^a^	0.3 ± 0.00^a^	0.28 ± 0.00^a^	0.58 ± 0.00^b^	0.55 ± 0.00^b^	0.28 ± 0.00^a^	0.28 ± 0.00^a^	0.59 ± 0.00^b^

*Bacillus subtilis* (Gram-positive)	MIC	<0.0005 ± 0.00^a^	0.44 ± 0.17^c,d^	0.53 ± 0.00^d^	0.46 ± 0.18^c,d^	0.3 ± 0.00^b,c^	0.14 ± 0.00^a,b^	0.29 ± 0.00^b,c^	0.14 ± 0.00^a,b^	0.14 ± 0.00^a,b^	0.28 ± 0.00^b,c^	0.30 ± 0.00^b,c,d^
	MBC	<0.0005 ± 0.00^a^	0.62 ± 0.08^d^	1.05 ± 0.00^e^	0.61 ± 0.00^d^	0.3 ± 0.00^c^	0.14 ± 0.00^b^	0.29 ± 0.00^c^	0.14 ± 0.00^b^	0.14 ± 0.00^b^	0.28 ± 0.00^c^	0.59 ± 0.00^d^

*Staphylococcus aureus* MRSA (Gram-positive)	MIC	0.14 ± 0.00^a^	0.58 ± 0.00^b,c^	1.05 ± 0.00^d^	0.86 ± 0.18^c,d^	0.45 ± 0.17^a,b^	0.42 ± 0.16^a,b^	0.44 ± 0.17^a,b^	0.41 ± 0.16^a,b^	0.42 ± 0.16^a,b^	0.56 ± 0.00^b,c^	0.30 ± 0.00^a,b^
	MBC	>0.25 ± 0.00^a^	0.84 ± 0.00^c,d^	2.14 ± 0.00^e^	1.06 ± 0.18^d^	0.45 ± 0.17^a,b^	0.88 ± 0.00^d^	0.44 ± 0.17^a,b^	0.41 ± 0.16^a,b^	0.56 ± 0.00^b,c^	0.56 ± 0.00^b,c^	0.30 ± 0.00^a,b^

*Salmonella typhimurium* (Gram-negative)	MIC	0.003 ± 0.001^a^	0.15 ± 0.00^b,c^	0.53 ± 0.00^e^	0.15 ± 0.00^b,c^	0.11 ± 0.04^a,b,c^	0.07 ± 0.00^a,b^	0.07 ± 0.00^a,b^	1.09 ± 0.00^f^	0.21 ± 0.08^c,d^	0.28 ± 0.00^d^	0.22 ± 0.09^c,d^
	MBC	0.016 ± 0.00^a^	0.30 ± 0.00^c^	1.20 ± 0.00^e^	0.28 ± 0.00^c^	0.29 ± 0.00^c^	0.14 ± 0.00^b^	0.14 ± 0.00^b^	1.56 ± 0.00^f^	0.22 ± 0.09^b,c^	0.56 ± 0.00^d^	0.30 ± 0.00^c^

*Enterococcus faecalis* (Gram-positive)	MIC	<0.0005 ± 0.00^a^	0.15 ± 0.00^b,c^	1.05 ± 0.00^f^	0.15 ± 0.00^b,c^	0.11 ± 0.04^a,b^	0.14 ± 0.00^a,b,c^	0.15 ± 0.00^b,c^	0.55 ± 0.00^e^	0.42 ± 0.16^d,e^	0.28 ± 0.00^c,d^	0.30 ± 0.00^b,c^
	MBC	0.01 ± 0.00^a^	0.58 ± 0.00^d^	1.05 ± 0.00^e^	0.15 ± 0.00^b^	0.15 ± 0.00^b^	0.14 ± 0.00^b^	0.15 ± 0.00^b^	0.55 ± 0.00^d^	0.56 ± 0.00^d^	0.28 ± 0.00^c^	0.30 ± 0.00^c^

*Staphylococcus aureus* (Gram-positive)	MIC	0.08 ± 0.01^a^	0.58 ± 0.00^a,b^	1.58 ± 0.61^c^	0.91 ± 0.35^b,c^	0.9 ± 0.35^a,b,c^	0.56 ± 0.00^a,b^	0.44 ± 0.17^a,b^	0.82 ± 0.32^a,b,c^	1.12 ± 0.00^b,c^	0.84 ± 0.32^a,b,c^	0.89 ± 0.34^a,b,c^
	MBC	0.08 ± 0.05^a^	0.58 ± 0.00^a,b^	2.11 ± 0.00^b,c^	0.91 ± 0.35^a,b,c^	0.9 ± 0.35^a,b,c^	0.56 ± 0.00^a,b^	1.45 ± 1.00^a,b,c^	2.73 ± 1.89^c^	1.12 ± 0.00^a,b,c^	1.12 ± 0.00^a,b,c^	2.49 ± 0.00^b,c^

*Pseudomonas aeruginosa* (Gram-negative)	MIC	>0.25	>5.12	>5.12	>5.12	>5.12	>5.12	>5.12	>5.12	>5.12	>5.12	>5.12
	MBC	>0.25	>5.12	>5.12	>5.12	>5.12	>5.12	>5.12	>5.12	>5.12	>5.12	>5.12

According to the homogeneity of variances using an ANOVA analysis, values in the same row with common letters are not statistically different (*P* > 0.05).

**Table 4:** Minimum inhibitory concentration (MIC, mg/ml) and minimum fungicidal concentration (MFC, mg/ml) values for *T. daenensis* essential oils from different ecotypes.

Fungi		Amphotericin (*μ*g/ml)	TD1	TD2	TD3	TD4	TD5	TD6	TD7	TD8	TD9	TD10
*Aspergillus niger*	MIC	0.82 ± 0.05^a^	0.08 ± 0.00^b^	0.08 ± 0.00^b^	0.10 ± 0.00^c^	0.08 ± 0.00^b^	0.08 ± 0.00^b^	0.08 ± 0.00^b^	0.08 ± 0.00^b^	0.08 ± 0.00^b^	0.08 ± 0.00^b^	0.08 ± 0.00^b^
	**MFC**	4.69 ± 1.80^a^	0.36 ± 0.00^c^	0.16 ± 0.00^b^	0.38 ± 0.00^c^	0.37 ± 0.00^c^	0.34 ± 0.00^c^	0.18 ± 0.00^b^	0.17 ± 0.00^b^	0.17 ± 0.00^b^	0.70 ± 0.00^d^	0.18 ± 0.00^b^

*Candida albicans*	**MIC**	0.40 ± 0.00^a^	0.58 ± 0.00^h^	0.52 ± 0.00^g^	0.30 ± 0.00^f^	0.30 ± 0.00^f^	0.13 ± 0.00^b^	0.29 ± 0.00^e,f^	0.27 ± 0.00^c^	0.27 ± 0.00^c,d^	0.28 ± 0.00^d,e^	0.29 ± 0.00^e,f^
	**MFC**	1.56 ± 0.02^a^	0.58 ± 0.00^h^	0.52 ± 0.00^g^	0.30 ± 0.00^f^	0.30 ± 0.00^f^	0.13 ± 0.00^b^	0.29 ± 0.00^e,f^	0.27 ± 0.00^c^	0.27 ± 0.00^c,d^	0.28 ± 0.00^d,e^	0.29 ± 0.00^e,f^

According to the homogeneity of variances using an ANOVA analysis, values in the same row with common letters are not statistically different (*P* > 0.05).

**Table 5:** *Thymus daenensis* ecotypic oil values for antioxidant and antigenotoxic activities.

Sample	VIT C	BHT	TD1	TD2	TD3	TD4	TD5	TD6	TD7	TD8	TD9	TD10
Bleaching inhibitory (BIC50 mg/ml)	0.01 ± 0.00^a^	0.03 ± 0.00^a,b^	0.08 ± 0.01^a,b^	0.08 ± 0.01^a,b^	0.05 ± 0.01^a,b^	0.02 ± 0.00^a,b^	1.06 ± 0.03^c^	0.01 ± 0.00^a^	0.04 ± 0.01^a,b^	0.18 ± 0.01^b^	0.03 ± 0.00^a,b^	0.04 ± 0.00^a,b^
Radical scavenging (SC_50_ mg/ml)	0.03 ± 0.00^a^	0.02 ± 0.00^a^	0.80 ± 0.05^e^	1.79 ± 0.11^g^	0.61 ± 0.03^c,d^	0.52 ± 0.01^b,c^	0.41 ± 0.01^b^	0.44 ± 0.02^b^	1.24 ± 0.05^f^	0.71 ± 0.03^d,e^	1.15 ± 0.05^f^	0.65 ± 0.05^c,d^
Comet inhibitory concentration (CIC_50_ mg/ml)	0.008 ± 0.00^a^	0.02 ± 0.00^a^	1.50 ± 0.32^b^	>5.00^e^	2.46 ± 1.00^b,c^	3.34 ± 0.70^c,d^	1.67 ± 0.47^b^	>5.00^e^	4.00 ± 0.50^e,d^	4.28 ± 0.61^c,d^	1.36 ± 0.54^a,b^	1.04 ± 0.13^a,b^

According to the homogeneity of variances using an ANOVA analysis, values in the same row with common letters are not statistically different (*P* > 0.05). The estimated CIC_50_ values for TD2 and TD6 are 7.78 and 5.68 mg/ml, respectively.

## Data Availability

The datasets generated and/or analyzed during the current study are available upon request to the corresponding author.

## Supplementary Materials

Supplementary Materials Supplementary Figure S1: antioxidant effects of *T. daenensis* essential oils base on DPPH assay. A fresh DPPH stock was performed and 2 milliliters of it was added to different concentrations of each compound (0–400 *μ*M) and placed in the dark for 30 min. Using ((control absorbance–sample absorbance) × 100/control absorbance) equation, scavenging capacity percentage was determined, where controls contained all reagents except the antioxidant factors. An essential oils concentration required for scavenge 50% of DPPH radicals is known as scavenging capacity-50 (SC50). Scavenging capacity-50 was calculated from the calibration curve determined by linear or nonlinear regression from the scavenging capacity percentage versus essential oils concentrations. Supplementary Figure S2: antioxidant effects of *T. daenensis* essential oils base on *β*-carotene bleaching assay. Bleaching assay was carried utilizing a *β*-carotene/linoleic acid emulsion technique. Briefly, 1 mg *β*-carotene, 40 mg of linoleic acid, and 200 mg of Tween-20 were homogenized in 2 ml of chloroform. Then, chloroform was rotary-evaporated at 40°C for 30 min and then 100 ml of oxygenated deionized water was combined with vigorous shaking to form a stable emulsion. Then, 2.5 ml of the emulsion was added to 350 *μ*l of different essential oils concentrations (0–400 *μ*M) and left at 50°C in the light for 2 h and optical density was monitored at 470 nm. Bleaching inhibition potential percentage was determined by [(sample absorbance at time 0 – sample absorbance after 2 h)  × 100/(control absorbance at time 0 – control absorbance after 2 h)] equation. Bleaching inhibitory capacity-50 (BIC50) is a concentration required for protection of half percentage of *β*-carotene molecules from bleaching and is calculated from the calibration curve determined by linear or nonlinear regression from the bleaching inhibition percentages versus alkaloid concentrations, where controls contained all reagents except the antioxidant factors. Supplementary Figure S3: the induced DNA damage by the *T. daenensis* essential oils was evaluated on lymphocytes cells by the COMET assay conducted under alkaline conditions. A serial dilution of each essential oil (0 to 600 *μ*M) was supplemented with 120 *μ* M H_2_O_2_ and stored for 5 min at room temperature. Then, 10000 cells were transferred to each dilution and the suspension was stored at 4°C for 30 min. Cells were harvested and sandwiched between two layers low-melting agarose on a slide. Cells were lysed and electrophoresed. Finally, DNA was stained with ethidium bromide and pictured using a fluorescent microscope (BX51; Tokyo, Japan). Representative COMET images are shown from (A) PBS (negative COMET), (B) H_2_O_2_-treated TD 9 TD 10 cells (positive COMET), and (C–H) simultaneous treatment with different concentration (0.01–5 mg/ml) for 30 min.
