# Supplementary material for: Ecotypic Variations Affected the Biological Effectiveness of Thymus daenensis Celak Essential Oil
Source: Evid Based Complement Alternat Med. 2021 Feb 9;2021:6686558. doi: 10.1155/2021/6686558 (PMC7889342; doi:10.1155/2021/6686558)
Supplement: Supplementary Materials — Supplementary Figure S1: antioxidant effects of T. daenensis essential oils base on DPPH assay. A fresh DPPH stock was performed and 2 milliliters of it was added to different concentrations of each compound (0–400 μM) and placed in the dark for 30 min. Using ((control absorbance–sample absorbance) × 100/control absorbance) equation, scavenging capacity percentage was determined, where controls contained all reagents except the antioxidant factors. An essential oils concentration required for scavenge 50% of DPPH radicals is known as scavenging capacity-50 (SC50). Scavenging capacity-50 was calculated from the calibration curve determined by linear or nonlinear regression from the scavenging capacity percentage versus essential oils concentrations. Supplementary Figure S2: antioxidant effects of T. daenensis essential oils base on β-carotene bleaching assay. Bleaching assay was carried utilizing a β-carotene/linoleic acid emulsion technique. Briefly, 1 mg β-carotene, 40 mg of linoleic acid, and 200 mg of Tween-20 were homogenized in 2 ml of chloroform. Then, chloroform was rotary-evaporated at 40°C for 30 min and then 100 ml of oxygenated deionized water was combined with vigorous shaking to form a stable emulsion. Then, 2.5 ml of the emulsion was added to 350 μl of different essential oils concentrations (0–400 μM) and left at 50°C in the light for 2 h and optical density was monitored at 470 nm. Bleaching inhibition potential percentage was determined by [(sample absorbance at time 0 – sample absorbance after 2 h) × 100/(control absorbance at time 0 – control absorbance after 2 h)] equation. Bleaching inhibitory capacity-50 (BIC50) is a concentration required for protection of half percentage of β-carotene molecules from bleaching and is calculated from the calibration curve determined by linear or nonlinear regression from the bleaching inhibition percentages versus alkaloid concentrations, where controls contained all reagents except the antioxidant factors. Sup [file 6686558.f1.pdf]

Ecotypic Variations Affected the Biological Effectiveness of *Thymus daenensis* Celak Essential Oil

Running title: Biological effectiveness of ecotypic thymus

Fatemeh Elahian[1], Maryam Garshasbi[2], Zahra Mehri Asiabar[2], Neda Gholamian Dehkordi[1], Alireza Yazdinezhad[2], Seyed Abbas Mirzaei[3,*]

[1] Department of Medical Biotechnology, School of Advanced Technologies, Shahrekord University of Medical Sciences, Shahrekord, Iran.

[2] Department of Pharmacognosy and Traditional Pharmaceuticals, School of Pharmacy, Zanjan University of Medical Sciences, Zanjan, Iran.

[3,*] Clinical Biochemistry Research Center, Basic Health Sciences Institute, Shahrekord University of Medical Sciences, Shahrekord, Iran.

[*]Author for Correspondence:
S.A. Mirzaei, PharmD, PhD
Clinical Biochemistry Research Center, Basic Health Sciences Institute,
Shahrekord University of Medical Sciences, Shahrekord, Iran.
Tel: (+98) 38 33331471
Fax: (+98) 38 33330709
E-mail: mirzaei.a@skums.ac.ir
dr_amirzaei@yahoo.com

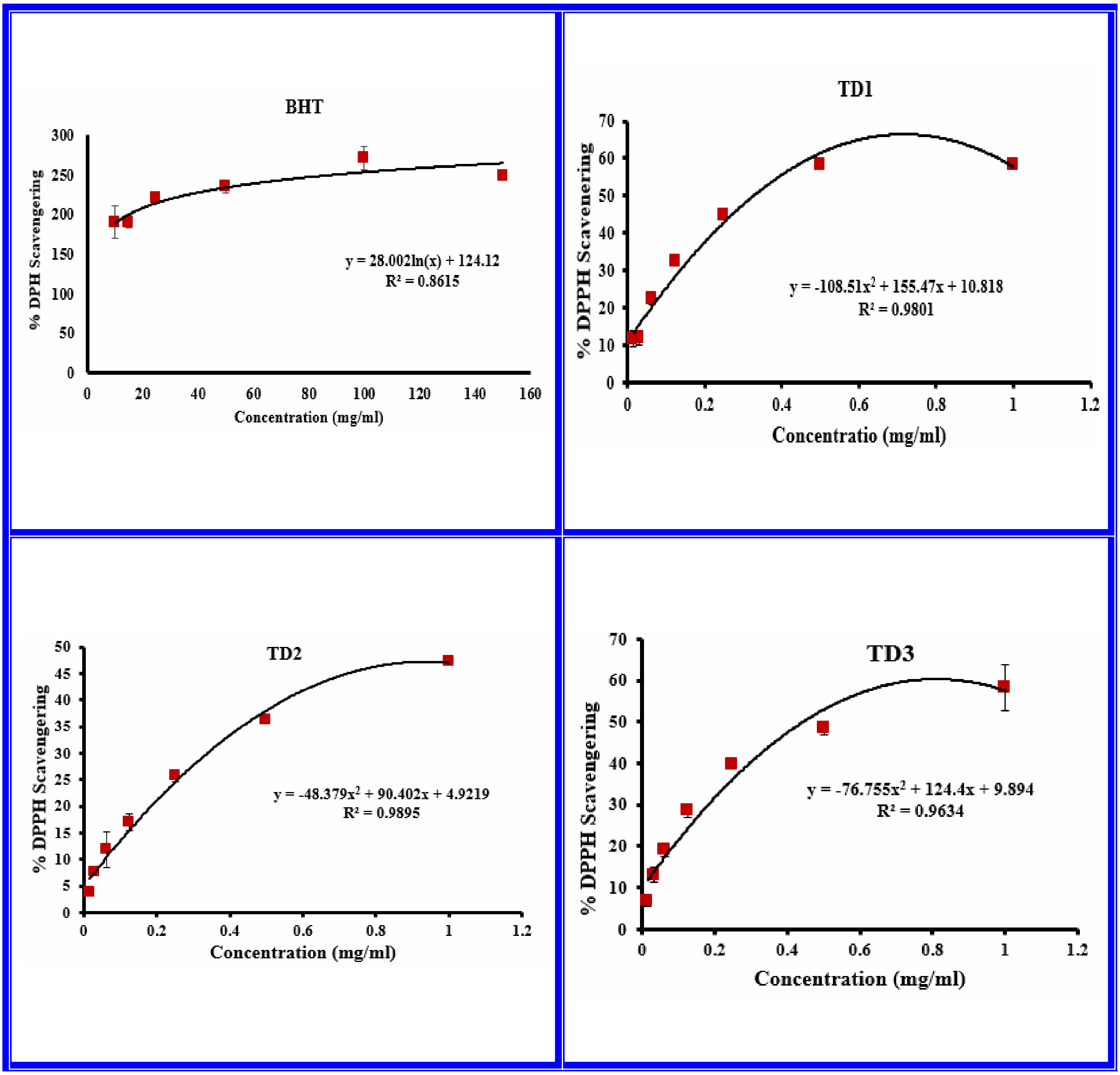
BHT
% DPPH Scavengering
300
250
200
150
100
50
0
0
20
40
60
80
100
120
140
160
Concentration (mg/ml)
y = 28.002ln(x) + 124.12
R² = 0.8615
TD1
% DPPH Scavenering
70
60
50
40
30
20
10
0
0
0.2
0.4
0.6
0.8
1
1.2
Concentratio (mg/ml)
y = -108.51x2 + 155.47x + 10.818
R² = 0.9801
TD2
% DPPH Scavengering
50
45
40
35
30
25
20
15
10
5
0
0
0.2
0.4
0.6
0.8
1
1.2
Concentration (mg/ml)
y = -48.379x2 + 90.402x + 4.9219
R² = 0.9895
TD3
% DPPH Scavengering
70
60
50
40
30
20
10
0
0
0.2
0.4
0.6
0.8
1
1.2
Concentration (mg/ml)
y = -76.755x2 + 124.4x + 9.894
R² = 0.9634

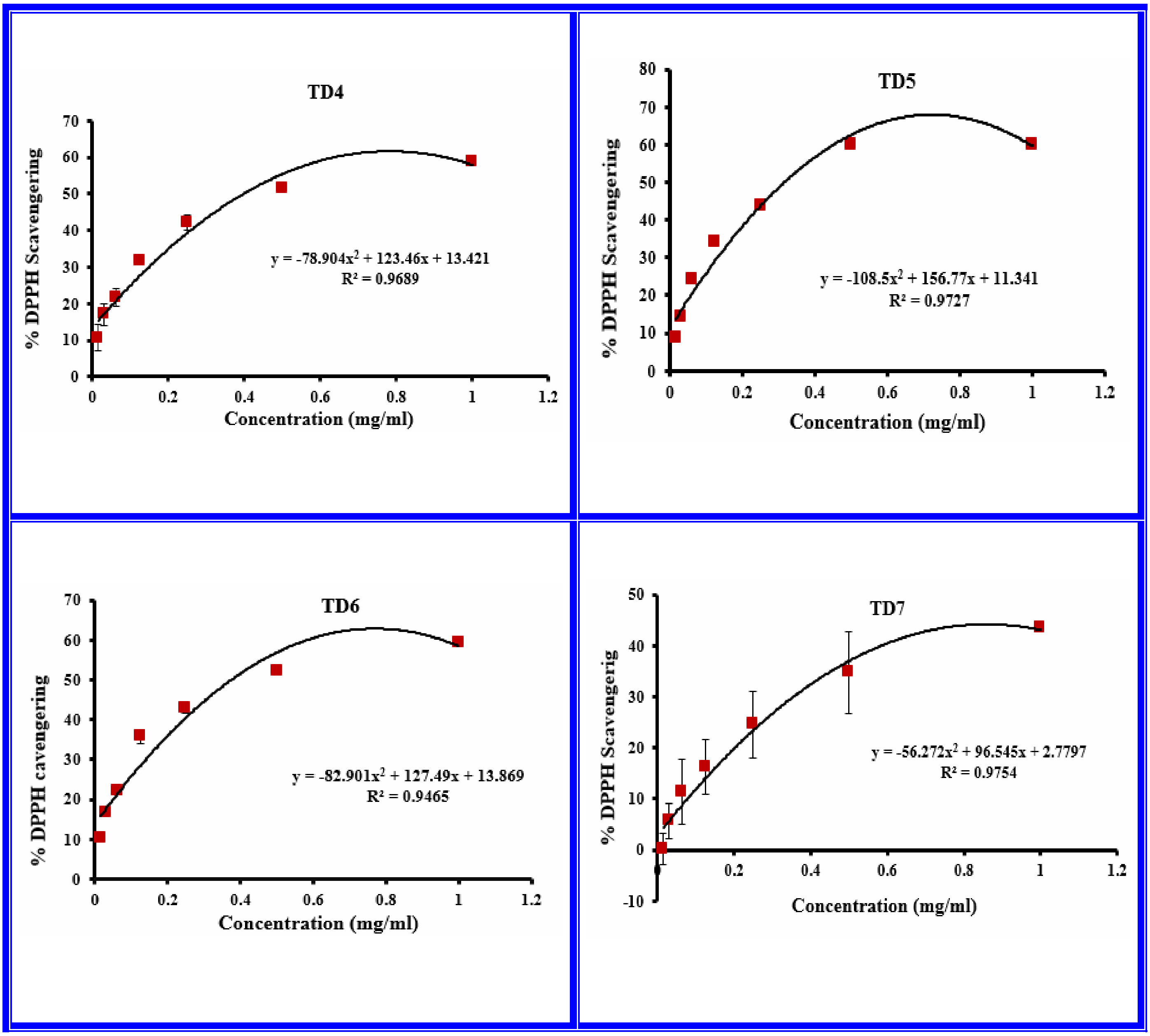
TD4
% DPPH Scavengering
Concentration (mg/ml)
y = -78.904x2 + 123.46x + 13.421
R² = 0.9689
TD5
% DPPH Scavengering
Concentration (mg/ml)
y = -108.5x2 + 156.77x + 11.341
R² = 0.9727
TD6
% DPPH cavengering
Concentration (mg/ml)
y = -82.901x2 + 127.49x + 13.869
R² = 0.9465
TD7
% DPPH Scavengerig
Concentration (mg/ml)
y = -56.272x2 + 96.545x + 2.7797
R² = 0.9754

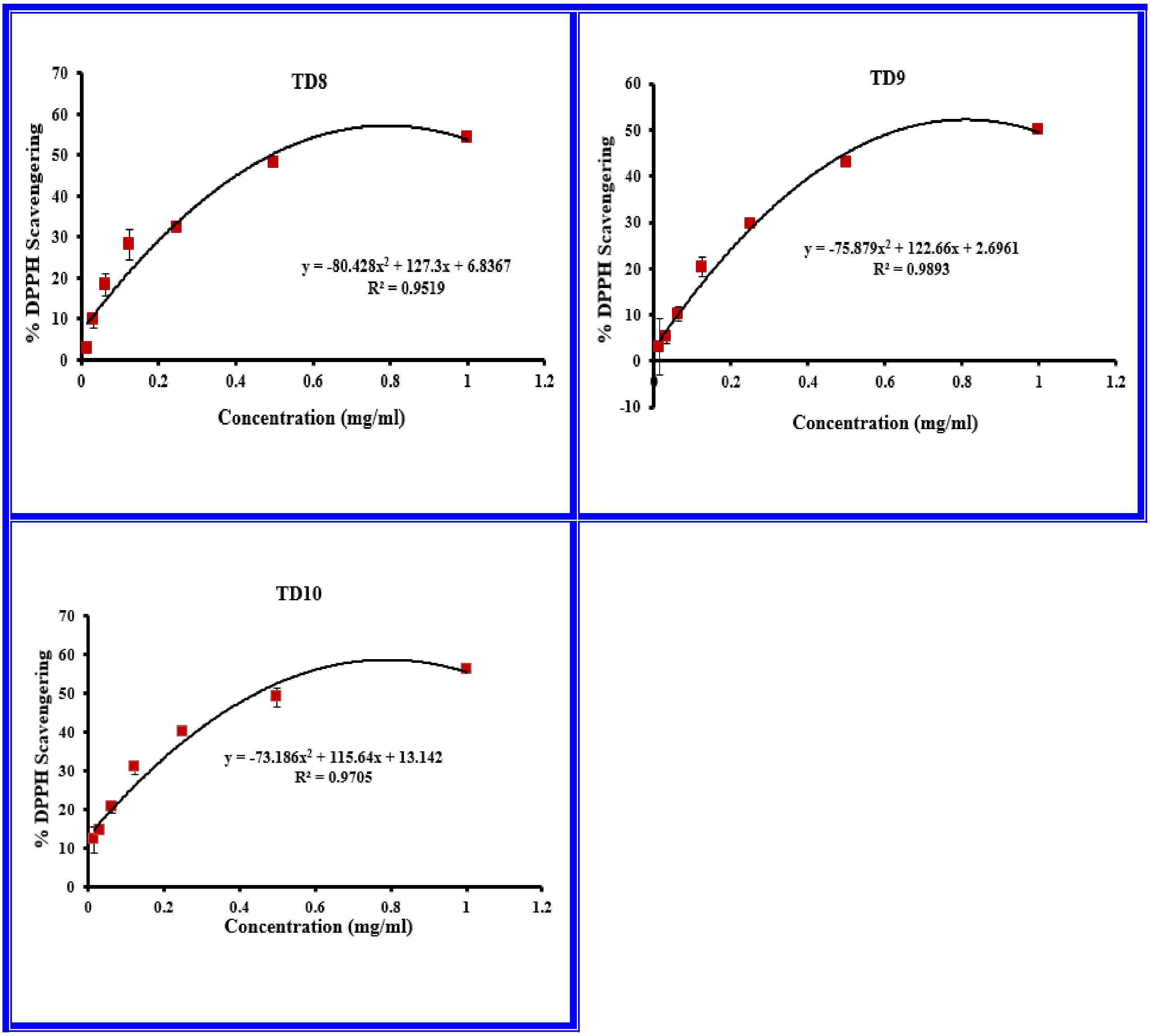

Supplementary Figure S1. Antioxidant effects of *T. daenensis* essential oils base on DPPH assay. A fresh DPPH stock was performed and 2 milliliter of it was added to different concentrations of each copmunds (0-400 μM) and placed in the dark for 30 min. Using [(control absorbance – sample absorbance) × 100 / control absorbance] equation, scavenging capacity percentage was determined. Where controls containing all reagents except the antioxidant factors. An essential oils concentration that required for scavenge 50 % of DPPH radicals is known as scavenging

capacity-50 (SC_{50}). Scavenging capacity-50 was calculated from the calibration curve determined by linear or non-linear regression from the scavenging capacity percentage versus essential oils concentrations.

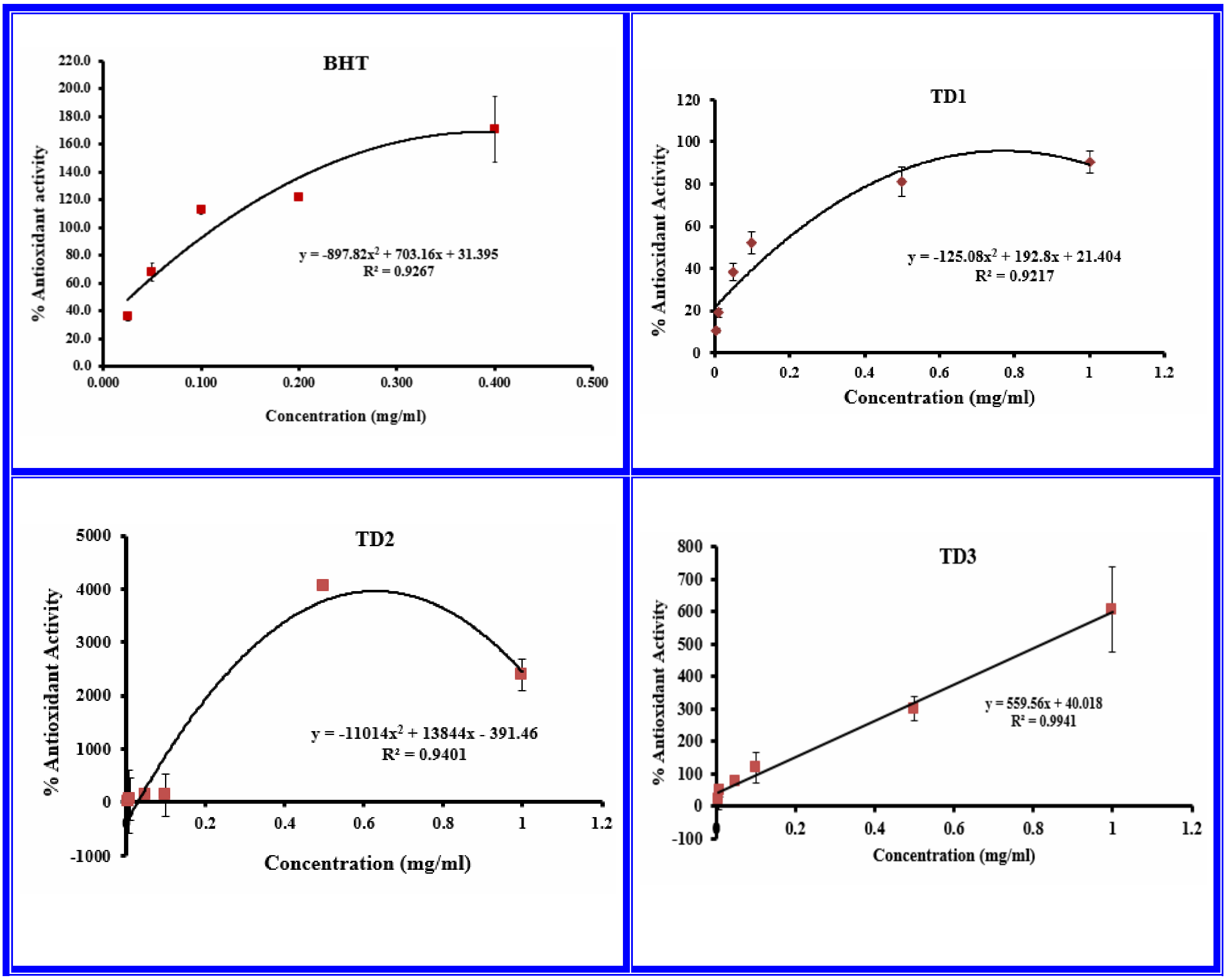
BHT
% Antioxidant activity
Concentration (mg/ml)
y = -897.82x2 + 703.16x + 31.395
R² = 0.9267
TD1
% Antioxidant Activity
Concentration (mg/ml)
y = -125.08x2 + 192.8x + 21.404
R² = 0.9217
TD2
% Antioxidant Activity
Concentration (mg/ml)
y = -11014x2 + 13844x - 391.46
R² = 0.9401
TD3
% Antioxidant Activity
Concentration (mg/ml)
y = 559.56x + 40.018
R² = 0.9941

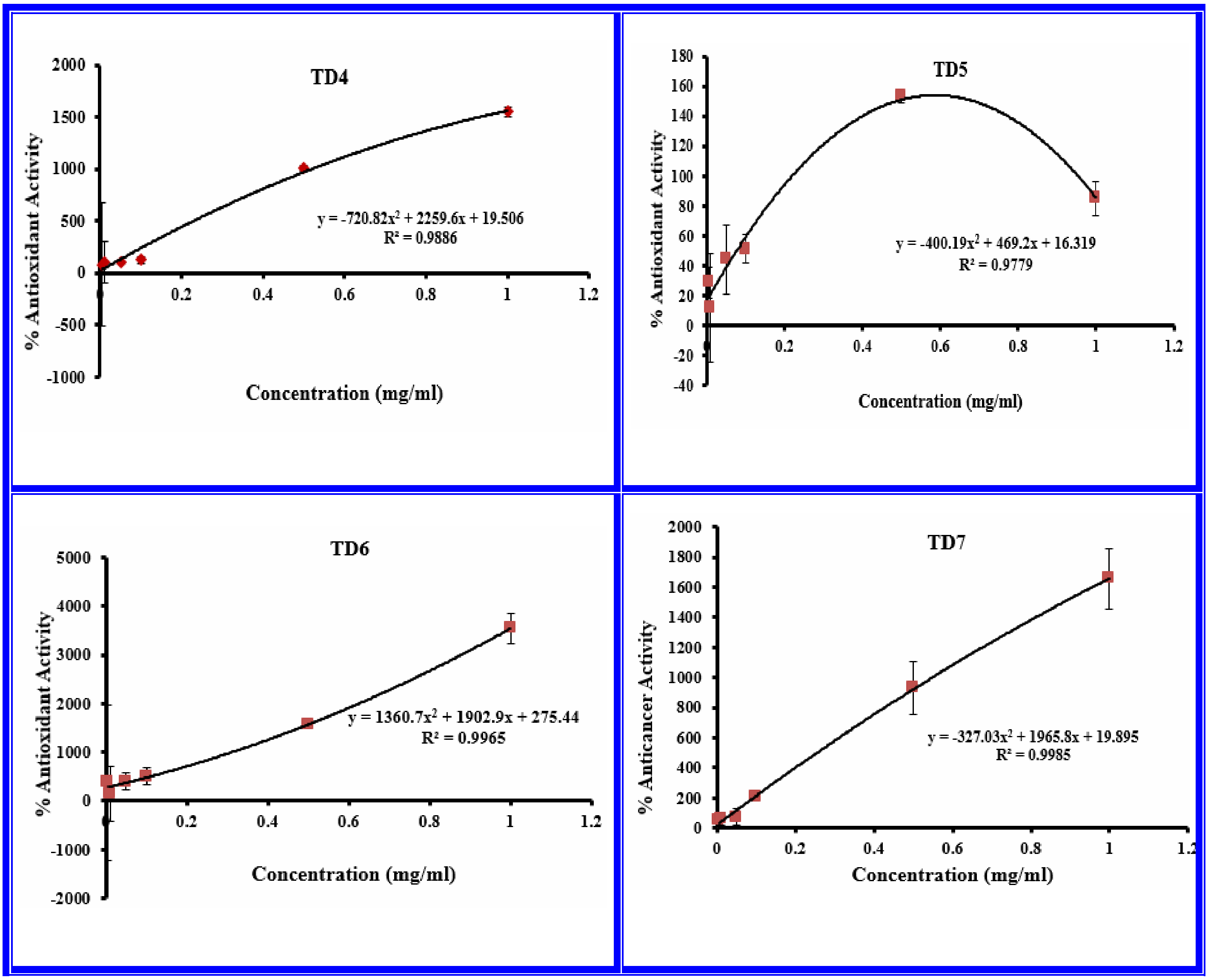
TD4
% Antioxidant Activity
2000
1500
1000
500
0
-500
-1000
0.2
0.4
0.6
0.8
1
1.2
$y = -720.82x^2 + 2259.6x + 19.506$
$R^2 = 0.9886$
Concentration (mg/ml)
TD5
% Antioxidant Activity
180
160
140
120
100
80
60
40
20
0
-20
-40
0.2
0.4
0.6
0.8
1
1.2
$y = -400.19x^2 + 469.2x + 16.319$
$R^2 = 0.9779$
Concentration (mg/ml)
TD6
% Antioxidant Activity
5000
4000
3000
2000
1000
0
-1000
-2000
0.2
0.4
0.6
0.8
1
1.2
$y = 1360.7x^2 + 1902.9x + 275.44$
$R^2 = 0.9965$
Concentration (mg/ml)
TD7
% Anticancer Activity
2000
1800
1600
1400
1200
1000
800
600
400
200
0
0
0.2
0.4
0.6
0.8
1
1.2
$y = -327.03x^2 + 1965.8x + 19.895$
$R^2 = 0.9985$
Concentration (mg/ml)

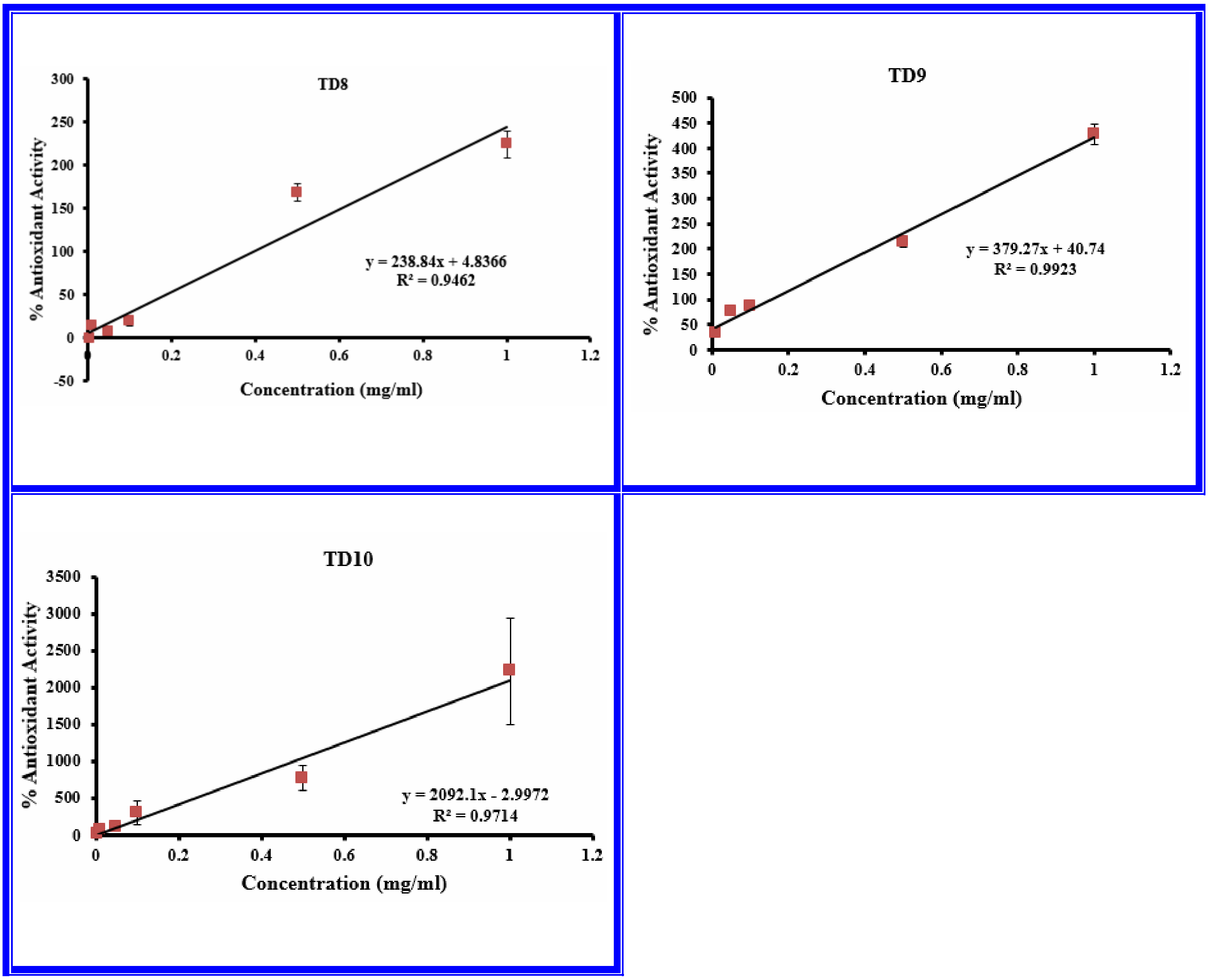

Supplementary Figure S2. Antioxidant effects of *T. daenensis* essential oils base on β-carotene bleaching assay. Bleaching assay was carried utilizing a β-carotene/linoleic acid emulsion technique. Briefly, 1 mg β-carotene, 40 mg of linoleic acid, and 200 mg of Tween-20 were homogenized in 2 ml of chloroform. Then chloroform was rotary evaporated at 40 °C for 30 min and then 100 ml of oxygenated deionized water was combined with vigorous shaking to form a stable emulsion. Then, 2.5 ml of the emulsion was added to 350 µl of different essential oils concentrations (0-400 µM) and left at 50 °C in the light for 2 h and optical density was monitored at 470 nm. Bleaching inhibition potential percentage was determined from [(sample absorbance at time 0 – sample absorbance after 2 h) × 100 / (control absorbance at time 0 –

control absorbance after 2 h)] equation. Bleaching inhibitory capacity-50 (BIC_{50}) is an concentration required for protection of half percentage of β-carotene molecules from bleaching and is calculated from the calibration curve determined by linear or non-linear regression from the bleaching inhibition percentages versus alkaloid concentrations. Where controls containing all reagents except the antioxidant factors.

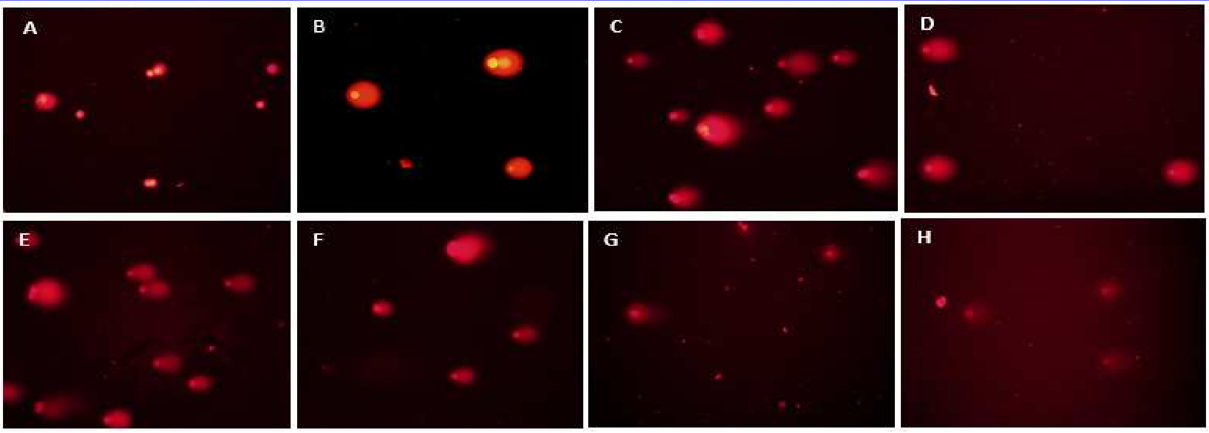

TD 1

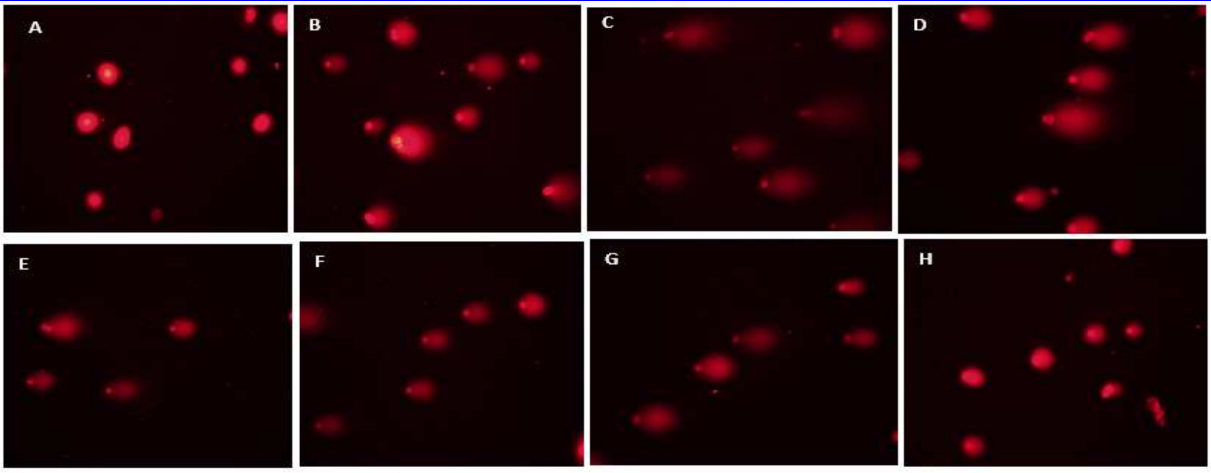

TD 2

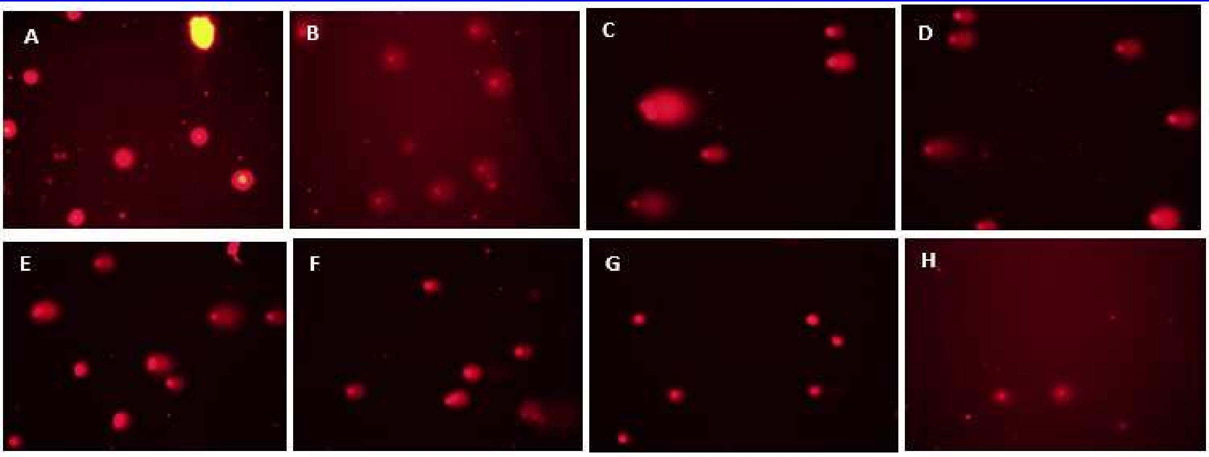

TD 3

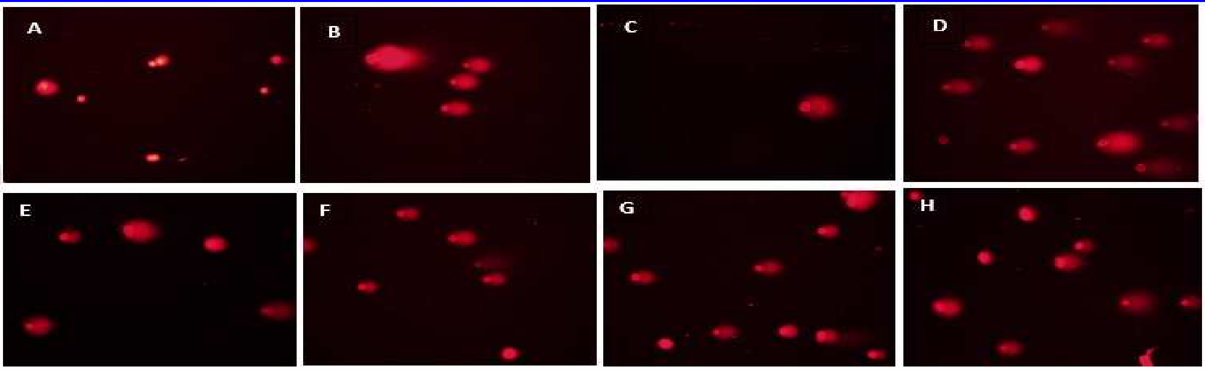

TD 4

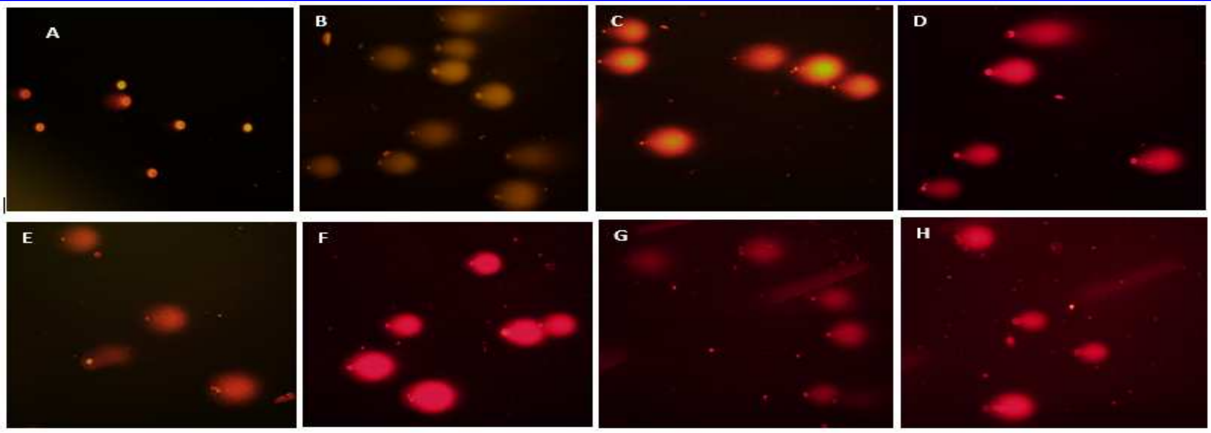

TD 5

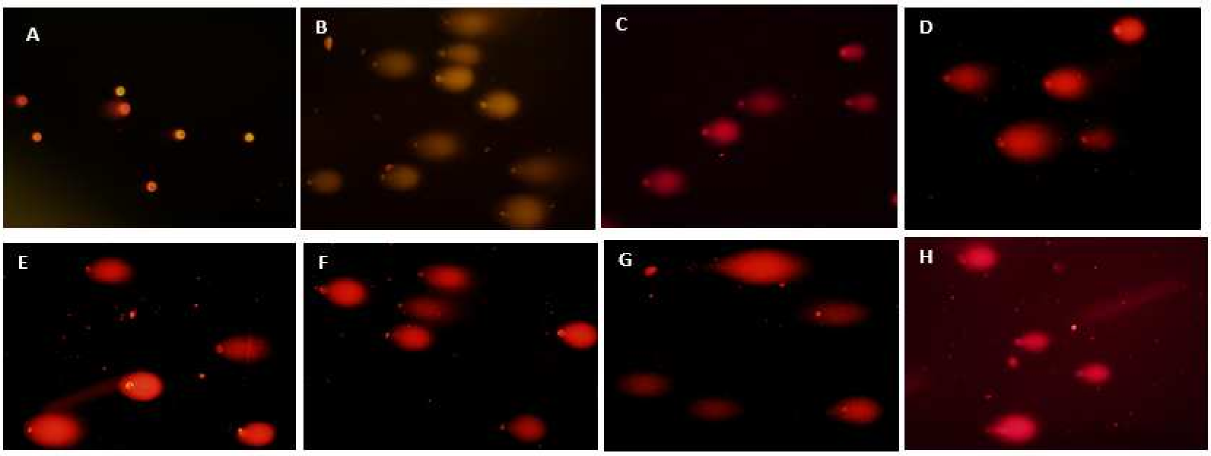

TD 6

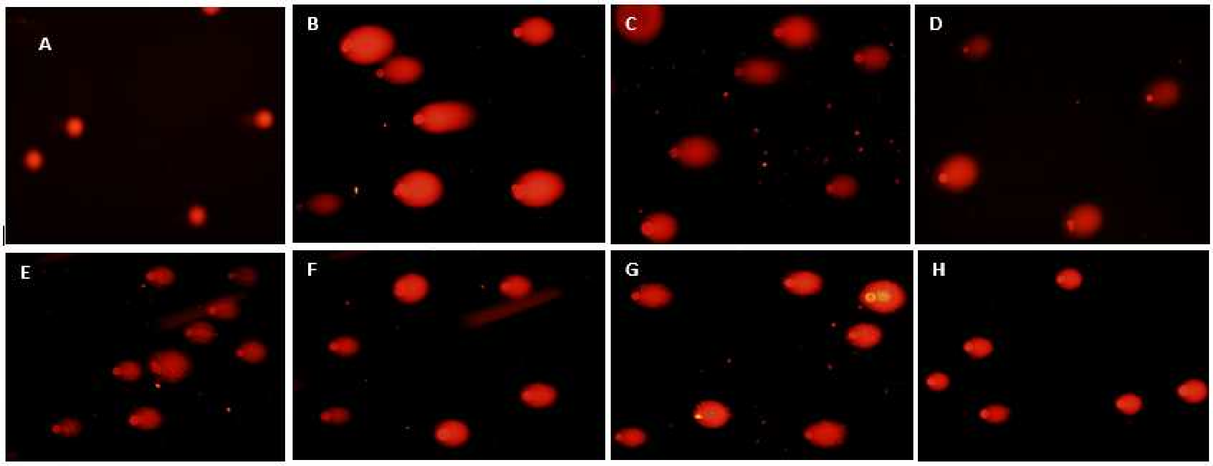

TD 7

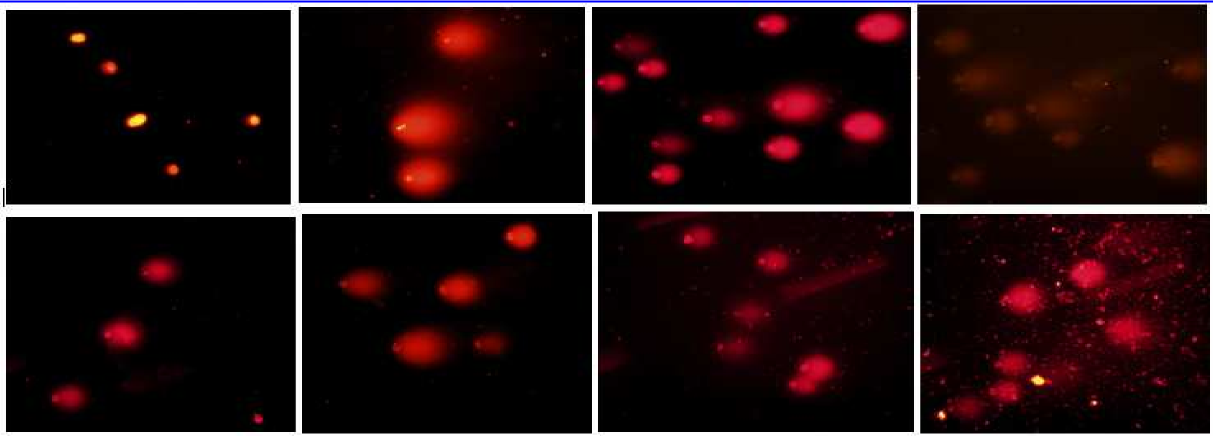

TD 8

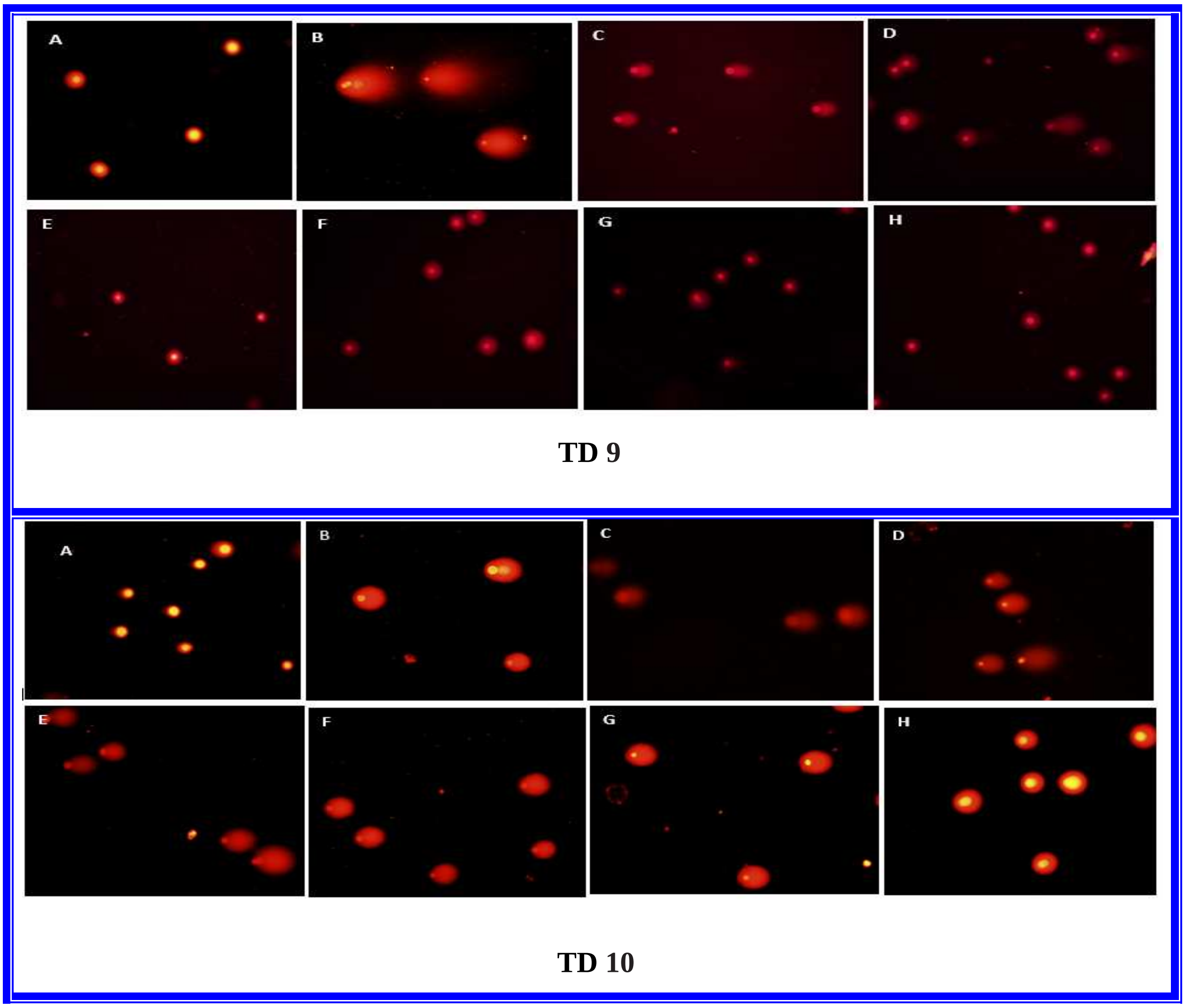

Supplementary Figure S3. The induced DNA damage by the *T. daenensis* essential oils was evaluated on Lymphocytes cells by the COMET assay conducted under alkaline conditions. A serial dilution of each essential oils (0 to 600 μM) was supplemented with 120 μM H_2O_2 and stored for 5 min at room temperature. Then, 10000 cells were transferred to each dilution and the suspension was stored at 4 °C for 30 min. Cells were harvested and sandwiched between two layers low melting agarose on a slide. Cells were lysed and electrophoresed. Finally, DNA were stained with ethidium bromide and pictured using a fluorescent microscope (BX51; Tokyo, Japan). Representative COMET-images from (A) PBS (negative COMET), (B) H_2O_2-treated

cells (positive COMET) and (C–H) simultaneous treatment with different concentration (0.01-5 mg/ml) for 30 min.
